# QTQTN motif upstream of the furin-cleavage site plays a key role in SARS-CoV-2 infection and pathogenesis

**DOI:** 10.1073/pnas.2205690119

**Published:** 2022-07-26

**Authors:** Michelle N. Vu, Kumari G. Lokugamage, Jessica A. Plante, Dionna Scharton, Aaron O. Bailey, Stephanea Sotcheff, Daniele M. Swetnam, Bryan A. Johnson, Craig Schindewolf, R. Elias Alvarado, Patricia A. Crocquet-Valdes, Kari Debbink, Scott C. Weaver, David H. Walker, William K. Russell, Andrew L. Routh, Kenneth S. Plante, Vineet D. Menachery

**Affiliations:** ^a^Department of Microbiology and Immunology, University of Texas Medical Branch, Galveston, TX 77555;; ^b^Institute for Human Infection and Immunity, University of Texas Medical Branch, Galveston, TX 77555;; ^c^World Reference Center of Emerging Viruses and Arboviruses, University of Texas Medical Branch, Galveston, TX 77555;; ^d^Department of Biochemistry and Molecular Biology, University of Texas Medical Branch, Galveston, TX 77555;; ^e^Institute for Translational Sciences, University of Texas Medical Branch, Galveston, TX;; ^f^Department of Pathology, University of Texas Medical Branch, Galveston, TX 77555;; ^g^Department of Molecular Microbiology and Immunology, Bloomberg School of Public Health, Johns Hopkins University, Baltimore, MD 21211;; ^h^Center for Biodefense and Emerging Infectious Disease, University of Texas Medical Branch, Galveston, TX 77555

**Keywords:** SARS-CoV-2, spike, glycosylation, QTQTN, furin cleavage site

## Abstract

The furin cleavage site (FCS), an unusual feature in the SARS-CoV-2 spike protein, has been spotlighted as a factor key to facilitating infection and pathogenesis by increasing spike processing. Similarly, the QTQTN motif directly upstream of the FCS is also an unusual feature for group 2B coronaviruses (CoVs). The QTQTN deletion has consistently been observed in in vitro cultured virus stocks and some clinical isolates. To determine whether the QTQTN motif is critical to SARS-CoV-2 replication and pathogenesis, we generated a mutant deleting the QTQTN motif (ΔQTQTN). Here, we report that the QTQTN deletion attenuates viral replication in respiratory cells in vitro and attenuates disease in vivo. The deletion results in a shortened, more rigid peptide loop that contains the FCS and is less accessible to host proteases, such as TMPRSS2. Thus, the deletion reduced the efficiency of spike processing and attenuates SARS-CoV-2 infection. Importantly, the QTQTN motif also contains residues that are glycosylated, and disruption of its glycosylation also attenuates virus replication in a TMPRSS2-dependent manner. Together, our results reveal that three aspects of the S1/S2 cleavage site—the FCS, loop length, and glycosylation—are required for efficient SARS-CoV-2 replication and pathogenesis.

## Significance

This study demonstrates that in addition to the furin cleavage site (FCS), both the length/composition of the exterior loop and glycosylation of the QTQTN motif are necessary for efficient SARS-CoV-2 infection and pathogenesis. Disruption of any of these three elements reduces SARS-CoV-2 replication, alters entry pathway utilization, and attenuates in vivo disease. Together, the work highlights the complexity of spike activation beyond just the presence of an FCS.

SARS-CoV-2 emerged in late 2019 and has caused the largest pandemic since the 1918 influenza outbreak (1). An unusual feature of SARS-CoV-2 is the presence of a furin cleavage site (FCS) in its spike protein (2). The CoV spike is a trimer of spike proteins composed of the S1 and S2 subunits, responsible for receptor binding and membrane fusion, respectively (1). After receptor binding, the spike protein is proteolytically cleaved at the S1/S2 and S2′ sites to activate the fusion machinery. For SARS-CoV-2, the spike protein contains a novel cleavage motif recognized by the host cell furin protease (PRRAR) directly upstream of the S1/S2 cleavage site that facilitates cleavage prior to virion release from the producer cell. This FCS, not found in other group 2B CoVs, plays a key role in spike processing, infectivity, and pathogenesis as shown by our group and others (3, 4).

Importantly, another novel amino acid motif, QTQTN, is found directly upstream of the FCS. This QTQTN motif, also absent in other group 2B CoVs, is often deleted and has been pervasive in cultured virus stocks of the alpha, beta, and delta variants (5–8). In addition, the QTQTN deletion has been observed in a small subset of patient samples as well (9–11). Because this deletion has been frequently identified, we set out to characterize it and determine whether it has consequences for viral replication and virulence. Using our infectious clone (12, 13), we demonstrated that the loss of this motif attenuates SARS-CoV-2 replication in respiratory cells in vitro and pathogenesis in hamsters. The QTQTN deletion results in reduced spike cleavage and diminished capacity to use serine proteases on the cell surface for entry. Importantly, mutations of glycosylation-enabling residues in the QTQTN motif results in similar replication attenuation despite intact spike processing. Together, our results highlight elements in the SARS-CoV-2 spike in addition to the FCS that contribute to increased replication and pathogenesis.

## Results

### ΔQTQTN Attenuates Viral Replication In Vitro.

**Fig. 1.**
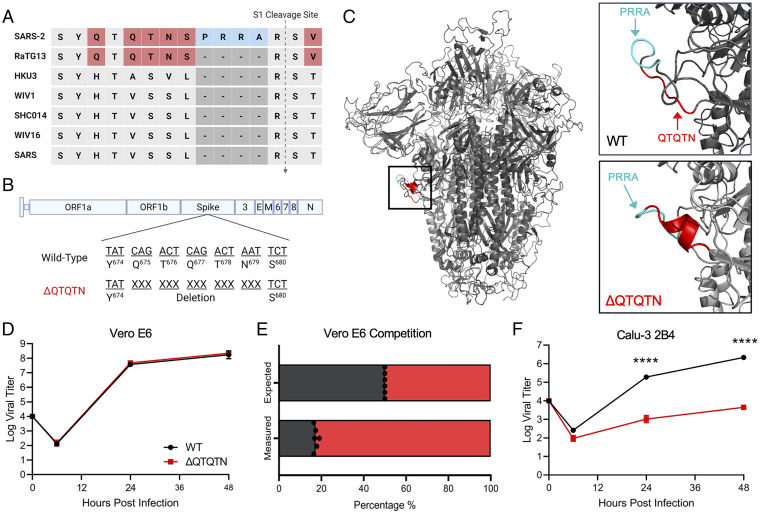
In vitro characterization of SARS-CoV-2 ΔQTQTN. (*A*) Comparison of S1/S2 cleavage site across SARS-CoV, SARS-CoV-2, and related bat CoVs. (*B*) Schematic of SARS-CoV-2 genome with deletion of QTQTN. (*C*) SARS-CoV-2 spike trimer (gray) with WT (*Upper*) and ΔQTQTN (*Lower*) overlaid. PRRA (cyan) is exposed with QTQTN (red) extending the loop (*Upper*). An α-helix is formed with deletion of QTQTN (red) and PRRA (cyan) is exposed (*Lower*). (*D*) Viral titer from Vero E6 infected with WT (black) or ΔQTQTN (red) SARS-CoV-2 at an MOI of 0.01 (*n* = 3). (*E*) Competition assay between WT and ΔQTQTN SARS-CoV-2 at a ratio of 1:1, showing RNA percentage from next-generation sequencing. (*F*) Viral titer from Calu-3 2B4 infected with WT or ΔQTQTN SARS-CoV-2 at an MOI of 0.01 (*n* = 3). Data are mean ± SD. Statistical analysis measured by two-tailed Student’s *t* test. *P ≤ 0.05; **P ≤ 0.01; ***P ≤ 0.001; ****P ≤ 0.0001 (*SI Appendix*, Fig. S1).

In addition to the FCS, comparison of group 2B coronavirus sequences also revealed the presence of an upstream QTQTN motif directly in the SARS-CoV-2 spike protein; this motif is absent in most other coronaviruses except for the closely related RaTG13 bat coronavirus (Fig. 1*A*). Importantly, this QTQTN motif is often deleted in SARS-CoV-2 strains propagated in Vero E6 cells (5–8). To explore the role of the QTQTN motif in SARS-CoV-2 infection and pathogenesis, we generated a mutant in the WA-1 background (early US case from 2020) by deleting QTQTN (ΔQTQTN) using our reverse genetics system (12, 13) (Fig. 1*B*). Examining the deletion on the SARS-CoV-2 spike structure, our modeling suggested that the ΔQTQTN mutant forms a stable α-helix in the loop containing the S1/S2 cleavage site (Fig. 1*C*). While the mutant retains the furin cleavage motif, its a-helix is predicted to make the loop less flexible and reduce access to the proteolytic cleavage site.

The deletion of QTQTN motif did not affect virus replication in Vero E6 cells (African green monkey kidney cells) with the rescue stock titer comparable to wild-type WA-1 (WT) in yield; yet, the ΔQTQTN mutant produced a large plaque morphology (*SI Appendix*, Fig. S1 *A* and *B*), as seen with a FCS knockout mutant (ΔPRRA) (3). We then evaluated replication kinetics of ΔQTQTN in Vero E6 cells and found no difference between WT and ΔQTQTN (Fig. 1*D*). However, following direct 1:1 competition infection, the ΔQTQTN mutant had a significant advantage relative to WT SARS-CoV-2 in Vero E6 cells (Fig. 1*E*). This fitness advantage for ΔQTQTN likely explains the accumulation of this mutation in Vero E6-amplified virus stocks, as we also observed emergence of this mutation in Vero E6 cells infected with WT alone (*SI Appendix*, Fig. S1 *C* and *D*). Notably, in Calu-3 2B4 cells, a human respiratory cell line, we observed a ∼2.5 log reduction in ΔQTQTN replication at both 24 and 48 h postinfection (hpi) (Fig. 1*F*). Together, the results indicate that ΔQTQTN mutant is attenuated in respiratory cells and has a fitness advantage in Vero E6 cells; these results are similar findings to those we reported for the SARS-CoV-2 FCS knockout virus (3).

### ΔQTQTN Attenuates Disease but Not Replication In Vivo.

**Fig. 2.**
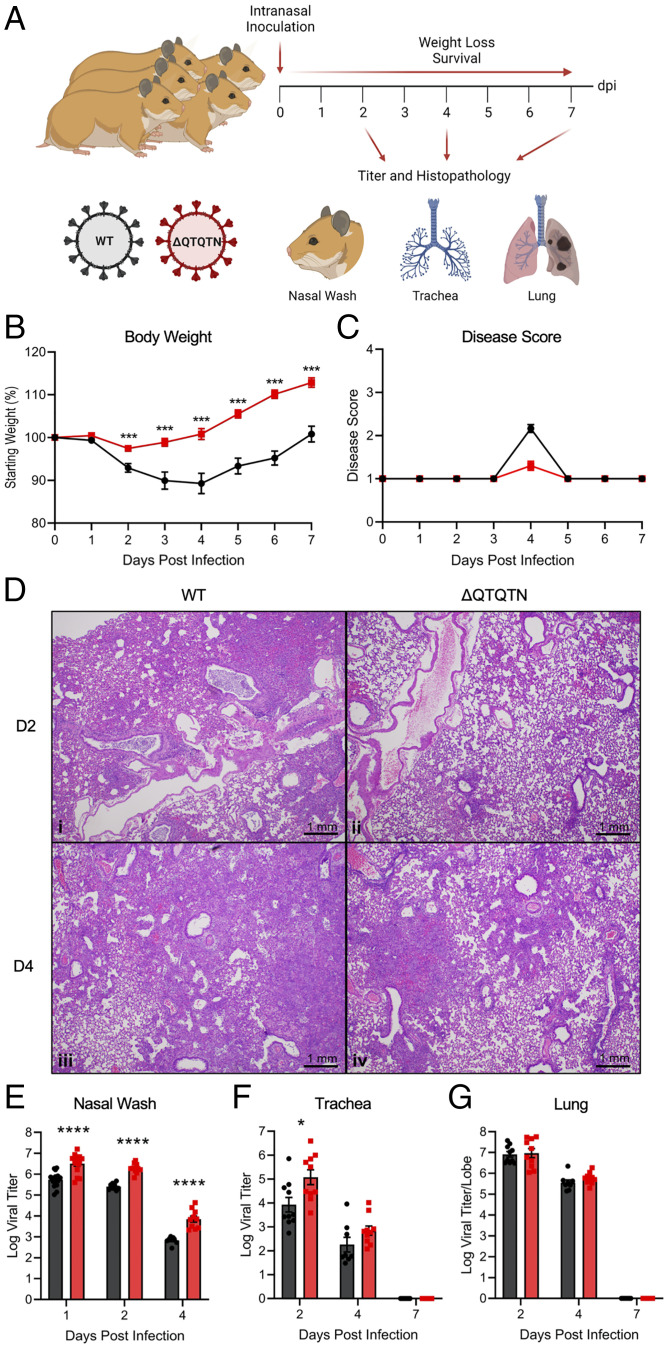
In vivo characterization of SARS-CoV-2 ΔQTQTN in golden Syrian hamsters. (*A*) Schematic of golden Syrian hamster infection with WT (black) or ΔQTQTN (red) SARS-CoV-2. (*B* and *C*) Three- to four-week-old male hamsters were infected with 105 pfu of WT or ΔQTQTN SARS-CoV-2 and monitored weight loss (*B*) and disease score (*C*) for 7 d. (*D*) Histopathology of hamster lungs manifested more extensive lesions in animals infected with WT SARS-CoV-2 on day 2 (*i*) (4×) than in animals infected with ΔQTQTN (*ii*) (4×). Lesions increased in volume on day 4 with greater proportions of the lungs affected in hamsters infected with WT (*iii*) (4×) than ΔQTQTN (iv) (4×) on day 4. (*E*–*G*) Viral titer was measured for nasal wash (*E*), trachea (*F*), and lung (*G*). Data are mean ± SEM. Statistical analysis measured by two-tailed Student’s *t* test. *P ≤ 0.05; **P ≤ 0.01; ***P ≤ 0.001; ****P ≤ 0.0001 (*SI Appendix*, Fig. S2).

We next evaluated the role of ΔQTQTN on virulence in an in vivo model. Three- to four-week-old male golden Syrian hamsters, which develop disease similar to that seen in humans (14), were intranasally inoculated with 10^5^ plaque-forming units (pfu) of WT SARS-CoV-2 or ΔQTQTN mutant and monitored for 7 d postinfection (dpi) (Fig. 2*A*). Hamsters infected with WT steadily lost weight from 2 dpi with average peak weight loss of ∼10% before beginning to recover at 5 dpi and regaining their starting weight by 7 dpi (Fig. 2*B*). The disease score peaks corresponded with maximum weight loss, with hamsters exhibiting ruffled fur, hunched posture, and/or reduced activity requiring additional welfare checks (Fig. 2*C*). In contrast, hamsters infected with ΔQTQTN experienced minimal weight loss, instead gaining weight over the course of the infection (Fig. 2*B*). Similarly, no obvious disease was observed in ΔQTQTN infected animals (Fig. 2*C*). Hamsters infected with ΔQTQTN developed pulmonary lesions that were less extensive than those in hamsters infected with WT SARS CoV-2, involving smaller portions of the infected lungs on both days 2 and 4 after intranasal inoculation (Fig. 2*D*). All of the lesions were similar, with interstitial pneumonia, peribronchitis, peribronchiolitis, and vasculitis with predominantly subendothelial and perivascular infiltration by lymphocytes and perivascular edema. Characteristic cytopathologic effects were observed in alveolar pneumocytes and bronchiolar epithelium, including cellular enlargement, binucleation and multinucleation, and prominent nucleoli. Together, the results demonstrate that the deletion of QTQTN motif attenuates SARS-CoV-2 disease in vivo.

Despite clear attenuation in disease, ΔQTQTN viral replication in vivo was not compromised compared to WT SARS-CoV-2. Indeed, ΔQTQTN viral titers were greater than WT SARS-CoV-2 with a 10-fold increase in nasal wash titers at 1, 2, and 4 dpi (Fig. 2*E*). Similar titer increases were observed in the trachea of infected hamsters at 2 dpi with equivalent titers at 4 dpi (Fig. 2*F*). Notably, viral titers were equivalent in the lungs for both 2 and 4 dpi (Fig. 2*G*). RNA expression data from hamster lung samples revealed clustering of WT and ΔQTQTN at 2 dpi and 4 dpi (*SI Appendix*, Fig. S2*A*). Of note, although more variability was present at 2 dpi, ΔQTQTN was slightly closer to mock samples at both time points. However, up-regulated genes were similar between WT and ΔQTQTN in comparison to mock at both time points (*SI Appendix*, Fig. S2 *B* and *C*). Together, these results indicate that attenuation of ΔQTQTN in vivo is not due to change in replication capacity. In addition, these data are consistent with in vivo results with the FCS knockout virus (3).

### ΔQTQTN Reduces Spike Processing and Entry.

**Fig. 3.**
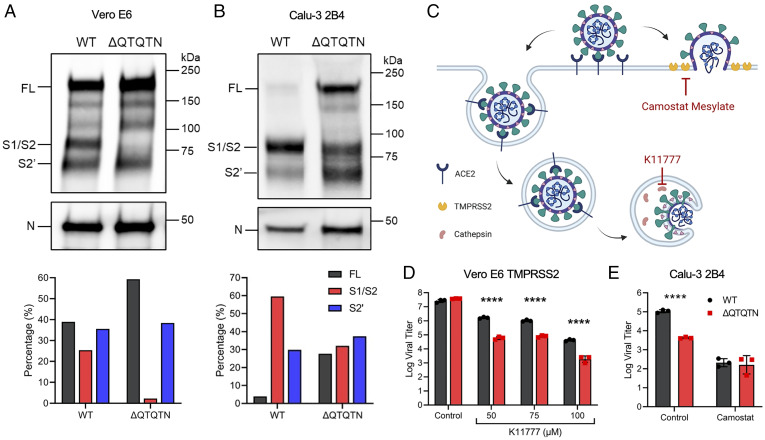
QTQTN motif is involved in spike processing and protease usage. (*A* and *B*) Purified WT and ΔQTQTN SARS-CoV-2 virions from Vero E6 (*A*) and Calu-3 2B4 (*B*) probed with anti-S or anti-N antibody (*Upper*). Full-length (FL), S1/S2 cleavage product, and S2′ cleavage product are indicated. Quantification of densitometry of FL (black), S1/S2 (red), and S2′ (blue) normalized to N shown (*Lower*). Results are representative of two experiments. (*C*) Schematic of SARS-CoV-2 entry and use of host proteases. Inhibitors for TMRPSS2 (camostat mesylate) and cathepsin (K11777) indicated. (*D*) Viral titer at 24 hpi from TMPRSS2-expressing Vero E6 pretreated with varying doses of cathepsin inhibitor K11777 and infected with WT (black) or ΔQTQTN SARS-CoV-2 (red) at an MOI of 0.01 (*n* = 3). (*E*) Viral titer at 24 hpi from Calu-3 2B4 pretreated with 100 µM of camostat mesylate and infected with WT or ΔQTQTN SARS-CoV-2 at an MOI of 0.01 (*n* = 3). Data are mean ± SD. Statistical analysis measured by two-tailed Student’s *t* test. *P ≤ 0.05; **P ≤ 0.01; ***P ≤ 0.001; ****P ≤ 0.0001 (*SI Appendix*, Fig. S3).

To examine the role of the QTQTN motif in spike processing, Vero E6 and Calu3-2B4 cells were infected with WT or ΔQTQTN and supernatant harvested at 24 hpi. Virions were then purified through sucrose cushion ultracentrifugation. Western blotting of the purified virions revealed reduced spike processing at the S1/S2 cleavage site for ΔQTQTN compared to WT in Vero E6 cells (Fig. 3*A*). Loss of the QTQTN motif resulted in little S1/S2 cleavage product and a significant increase in full-length spike compared to WT control. A similar reduction in spike processing was seen in Calu3-2B4 cells, although with more processing overall compared to in Vero E6 (Fig. 3*B*). Thus, deletion of the QTQTN motif impairs spike cleavage at the S1/S2 site, similar to findings with the SARS-CoV-2 mutants lacking the FCS (3, 4).

After receptor binding, the CoV spike protein is cleaved by a host protease as part of the virus entry process. Different proteases can be utilized to activate the spike fusion machinery: serine proteases like TMPRSS2 at the cell surface or cathepsins within endosomes (Fig. 3*C*). Our prior work found that the absence of TMPRSS2 in Vero E6 cells plays a role in selection of SARS-CoV-2 strains with FCS deletions (3). Calu-3 2B4 cells also express high levels of TMPRSS2. We therefore hypothesized that the absence of TMPRSS2 activity contributes to ΔQTQTN selection in Vero E6 and attenuation in Calu3 2B-4 cells. To test this hypothesis, Vero E6 cells expressing TMPRSS2 were pretreated with cathepsin inhibitor K11777 before infection with WT or ΔQTQTN, and viral titers were measured at 24 hpi. With cathepsin inhibited and TMPRSS2 activity intact, a significant, ∼1.5 log reduction in viral titer was observed for ΔQTQTN compared to WT over a dose range of K11777, mirroring the attenuation observed in the Calu-3 2B4 cells (Fig. 3*D*). Infection of untreated TMPRSS2-expressing Vero E6 revealed no difference in replication between WT and ΔQTQTN (*SI Appendix*, Fig. S3). When Calu-3 2B4 cells were pretreated with the serine protease inhibitor camostat mesylate, WT SARS-CoV-2 titers were reduced and equivalent to ΔQTQTN (Fig. 3*E*). Together, these data indicate that the loss of the QTQTN motif reduces the capacity of the virus to use TMPRSS2 for entry.

### Glycosylation of the QTQTN Motif Contributes to Spike Processing.

**Fig. 4.**
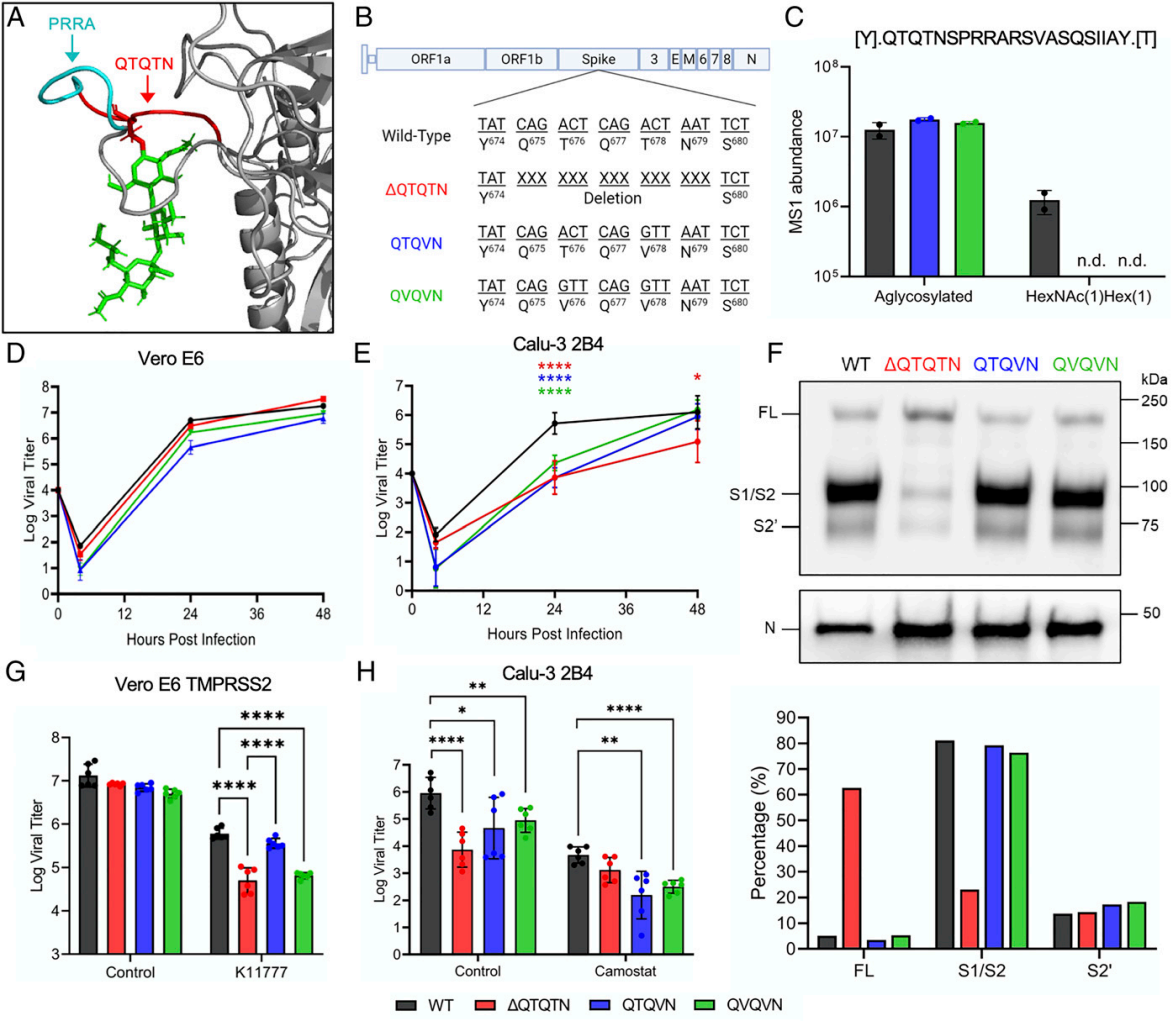
Glycosylation of QTQTN motif contributes to SARS-CoV-2 virulence. (*A*) Structural modeling of O-linked glycosylation on threonine 678 (red) of QTQTN motif. PRRA (cyan) remains exposed. (*B*) Schematic of SARS-CoV-2 genome with glycosylation mutations. (*C*) MS1 label-free abundance of WT (black), QTQVN (blue), and QVQVN (green) SARS-CoV-2 spike chymotrypsin peptide Q675-Y975 from sucrose cushion-purified virions (*SI Appendix*, Fig. S4). n.d., not detected. (*D*) Viral titer from Vero E6 infected with WT (black), ΔQTQTN (red), QTQVN (blue), or QVQVN (green) SARS-CoV-2 at an MOI of 0.01 (*n* = 3). (*E*) Viral titer from Calu-3 2B4 infected with WT, ΔQTQTN, QTQVN, or QVQVN SARS-CoV-2 at an MOI of 0.01 (*n* = 6). (*F*) Purified WT, ΔQTQTN, QTQVN, and QVQVN SARS-CoV-2 virions from Calu-3 2B4 probed with anti-S or anti-N antibody. Full-length (FL), S1/S2 cleavage product, and S2′ cleavage product are indicated. Results are representative of two experiments (*SI Appendix*, Fig. S5). (*G*) Viral titer at 24 hpi from TMPRSS2-expressing Vero E6 pretreated with 50 µM of K11777 and infected with WT, ΔQTQTN, QTQVN, or QVQVN SARS-CoV-2 at an MOI of 0.01 (*n* = 6). (*H*) Viral titer at 24 hpi from Calu-3 2B4 pretreated with 50 µM of camostat mesylate and infected with WT, ΔQTQTN, QTQVN, or QVQVN SARS-CoV-2 at an MOI of 0.01 (*n* = 6). Data are mean ± SD. Statistical analysis measured by two-tailed Student’s *t* test. *P ≤ 0.05; **P ≤ 0.01; ***P ≤ 0.001; ****P ≤ 0.0001.

As the absence of the QTQTN motif attenuates SARS-CoV-2, we set out to determine if the QTQTN motif itself has a significant role during infection. Notably, the second threonine, T678, of the motif has been previously shown to be O-linked glycosylated (15, 16). Structurally, the QTQTN site resides on an exterior loop of the spike and is capable of accommodating large glycans, which may contribute to interactions with proteases like TMPRSS2 (Fig. 4*A*). To determine the role of glycosylation in spike processing, we generated mutants abolishing the glycosylated T678 (QTQVN) alone or together with the first threonine T676 (QVQVN) to exclude possible compensatory glycosylation (Fig. 4*B*). To confirm the mutations disrupted glycosylation of the QTQTN motif, virions from Vero E6-produced supernatant were purified through sucrose cushion and prepared for liquid chromatography-tandem mass spectrometry analysis. Peptide fragments were analyzed for posttranslational modifications, including *N*-linked and O-linked glycosylation. Similar to other studies, T678 of WT spike was detected to have 10% occupancy with O-glycan HexNAc(1)Hex (1) (Fig. 4*C* and *SI Appendix*, Fig. S4 *A* and *B*). In contrast, no O-linked glycosylation or other modification was detected across the S1/S2 cleavage site of QTQVN or QVQVN mutants (Fig. 4*C* and *SI Appendix*, Fig. S4 *C* and *D*).

Similar to ΔQTQTN, the glycosylation mutations did not affect virus yield with titers comparable to WT (*SI Appendix*, Fig. S5*A*). However, plaque morphologies of QTQVN and QVQVN were more similar to WT than to the ΔQTQTN mutant (*SI Appendix*, Fig. S5*B*). Viral replication in Vero E6 cells was not affected for QTQVN and QVQVN mutants; however, both were attenuated at 24 hpi in Calu-3 2B4 cells, mirroring what was observed with the ΔQTQTN mutant, before reaching equivalent endpoint titer as to WT by 48 hpi (Fig. 4 *D* and *E*). Together, these results indicate that the loss of glycosylation sites in the QTQTN motif attenuates replication in Calu-3 2B4 cells.

We next examined if the glycosylation-defective mutants had altered spike processing similar to ΔQTQTN. Western blotting of virions purified from Calu-3 2B4 cells by sucrose cushion ultracentrifugation revealed intact spike processing at the S1/S2 site with both glycosylation mutants (Fig. 4*F*). Contrasting with ΔQTQTN, the QTQVN and QVQVN mutants had significant S1/S2 spike cleavage with levels similar to WT SARS-CoV-2. Likewise, spike processing of QTQVN and QVQVN in normal and TMPRSS2-expressing Vero E6 cells was similar to that of WT (*SI Appendix*, Fig. S5 *C* and *D*). Together, these results suggest that the loss of glycosylated residues does not impact spike processing of SARS-CoV-2.

To further understand the role of glycosylation on the QTQTN motif, we next examined if glycosylation is involved in protease interaction and usage. Using TMPRSS2-expressing Vero E6, we pretreated cells with K11777 to disrupt cathepsin activity, infected with WT, ΔQTQTN, QTQVN, or QVQVN, and examined viral titers at 24 hpi (Fig. 4*G*). While the ΔQTQTN titer was reduced compared to WT as observed before, the QTQVN titer was equivalent to WT (Fig. 4*G*). In contrast, disruption of both glycosylation residues with the QVQVN mutant titer resulted in attenuation equivalent to that of ΔQTQTN, suggesting that abolishing both O-linked glycosylation sites disrupted TMPRSS2 utilization (Fig. 4*G*). We subsequently pretreated Calu-3 2B4 cells with camostat mesylate to disrupt serine protease activity and infected with the glycosylation mutants (Fig. 4*H*). Interestingly, while treatment with camostat in Calu-3 2B4 cells reduced WT titer to equivalent levels as ΔQTQTN, viral titers of QTQVN and QVQVN mutants were even lower. However, the overall differences in titer between WT and the glycosylation mutants was reduced, suggesting that glycosylation is important for TMPRSS2 utilization and entry (Fig. 4*H*). Overall, these results argue that glycosylation of the QTQTN motif is important to protease interactions with spike and SARS-CoV-2 infection.

### The QTQTN Motif Is Necessary for Efficient SARS-CoV-2 Infection and Pathogenesis.

**Fig. 5.**
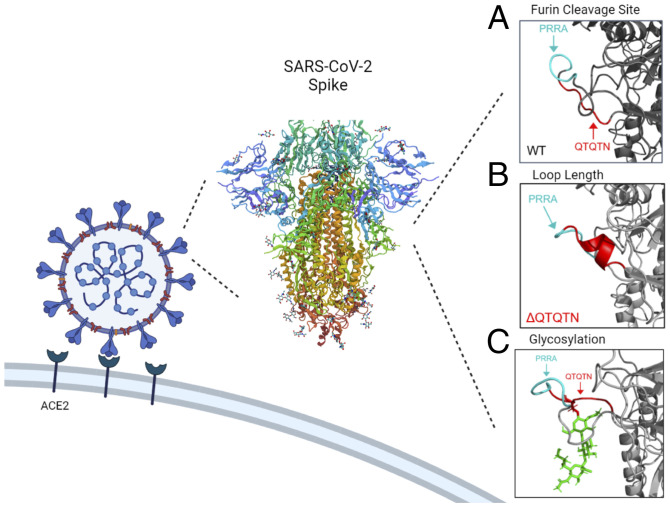
Characteristics of SARS-CoV-2 S1/S2 cleavage site for efficient infection. The SARS-CoV-2 S1 cleavage site contains multiple components required for efficient infection and virulence: the FCS, PRRA, is important for spike processing (*A*); the loop length/composition manages FCS accessibility and protease interaction (*B*); and glycosylation is involved in protease interaction (*C*). SARS-CoV-2 spike protein (18).

The presence of the FCS in SARS-CoV-2 plays a critical role in infection and pathogenesis by facilitating an increase spike processing upon nascent virion release (3). This FCS, unusual to SARS-like coronaviruses, has been highlighted as a potential “smoking gun” for an engineered virus (17). Yet, the FCS alone is insufficient to drive infection and pathogenesis. The upstream QTQTN motif adds two distinct elements that contribute to this capacity and virulence. The loss of the QTQTN motif produces a shorter, more rigid exterior loop in the spike, likely reducing access to the FCS (18). The result is a significant reduction in spike processing and attenuation of the ΔQTQTN mutant both in vitro and in vivo. Additionally, while mutations of the glycosylation residues in the QTQTN motif do not change overall spike processing, the modification of the motif attenuates virus replication in a TMPRSS2-dependent manner. Similar TMPRSS2-dependent attenuation has been observed in pseudoviruses displaying ΔQTQTN SARS-CoV-2 spike protein (19). Overall, our results argue that the FCS, the length/composition of the exterior loop, and glycosylation of the QTQTN motif are all needed for efficient infection and pathogenesis (Fig. 5). Disruption of any of these three elements attenuates SARS-CoV-2, highlighting the complexity of spike activation beyond the simple presence of an FCS .

## Methods

### Cell Culture.

Vero E6 cells were grown in Dulbecco modified Eagle medium (DMEM; Gibco #11965–092) supplemented with 10% fetal bovine serum (FBS) (HyClone #SH30071.03) and 1% antibiotic-antimycotic (Gibco #5240062). Calu-3 2B4 cells were grown in DMEM supplemented with 10% defined FBS (HyClone #SH30070.03), 1% antibiotic-antimycotic, and 1 mg/mL sodium pyruvate. Vero E6 expressing TMPRSS2 cells were grown in DMEM (Gibco #11885–084) supplemented with 10% FBS and 1 mg/mL geneticin (Gibco #10131035).

### Viruses.

The recombinant WT and mutant SARS-CoV-2 virus sequences are based on the USA-WA1/2020 isolate sequence provided by the World Reference Center for Emerging Viruses and Arboviruses (WRCEVA), which was originally obtained from the US Centers for Disease Control and Prevention (CDC) as previously described (20). The mutant viruses (ΔQTQTN, QTQVN, and QVQVN) were generated using standard cloning techniques and reverse genetics system as previously described (3, 12). Mutations were verified through Sanger sequencing of cDNA produced from viral RNA extract. Standard plaque assays were used for virus titer.

### In Vitro Infection.

Viral infections in Vero E6, Calu-3 2B4 and TMRPSS2-expressing Vero E6 cells were performed as previously described (13, 21). Briefly, cells were washed with phosphate buffered saline (PBS) and infected with WT or mutant SARS-CoV-2 at an MOI of 0.01 for 45 min at 37 °C. Following absorption, cells were washed three times with PBS and fresh growth media was added. Three or more biological replicates were collected at each time point.

### Protease Inhibitor Treatment.

TMPRSS2-expressing Vero E6 or Calu-3 2B4 cells were pretreated with 50 to 100 µM of K11777 (AdipoGen #AG-CR1-0158-M005) or 50 to 100 µM of camostat mesylate (Sigma-Aldrich #SML0057-10MG), respectively, in 1 mL growth medium for 1 h at 37 °C. Cells were subsequently washed with PBS and infected with WT or mutant SARS-CoV-2 at an MOI of 0.01 as described in *In vitro Infection*.

### Competition Assay and Next-Generation Sequencing Analysis.

Ratios (1:0, 1:1, and 0:1 WT:ΔQTQTN) for the competition assays were determined by plaque-forming unit of virus stock. Vero E6 cells were infected with a total multiplicity of infection (MOI) of 0.01 (WT alone, 1:1 WT:ΔQTQTN, or ΔQTQTN alone) as described in *In vitro Infection*. RNA was collected from cell lysate with TRIzol reagent (Invitrogen #15596018) and extracted with Direct-zol RNA Miniprep Plus kit (Zymo #R2072). RNA libraries were prepared by ClickSeq and sequenced as previously described (3, 22).

### Virion Purification and Western Blotting.

Vero E6, Calu-3 2B4, and TMPRSS2-expressing Vero E6 cells were infected with WT or mutant SARS-CoV-2 at an MOI of 0.01. Culture supernatant was harvested 24 hpi and clarified by low-speed centrifugation. Virus particles were then pelleted by ultracentrifugation through a 20% sucrose cushion at 26,000 rpm for 3 h using a Beckman SW28 rotor. Pellets were resuspended in 2× Laemmli buffer to obtain protein lysates. Relative viral protein levels were determined by sodium dodecyl sulfate-polyacrylamide gel electrophoresis (SDS-PAGE) followed by Western blot analysis as previously described (3, 20, 23, 24). In brief, sucrose-purified WT and mutant SARS-CoV-2 virions were inactivated by boiling in Laemmeli buffer. Samples were loaded in equal volumes into 4 to 20% Mini-PROTEAN TGX Gels (Bio-Rad #4561093) and electrophoresed by SDS–PAGE. Protein was transferred to polyvinylidene difluoride (PVDF) membranes. Membranes were probed with SARS-CoV S-specific antibodies (Novus Biologicals #NB100-56578) and followed with horseradish peroxidase (HRP)-conjugated anti-rabbit antibody (Cell Signaling Technology #7074S). Membranes were stripped and reprobed with SARS-CoV *N*-specific antibodies (provided by S. Makino) and the HRP-conjugated anti-rabbit secondary IgG to measure loading. Signal was developed using Clarity Western ECL substrate (Bio-Rad #1705060) or Clarity Max Western ECL substrate (Bio-Rad #1705062) and imaging on a ChemiDoc MP System (Bio-Rad #12003154). Densitometry was performed using ImageLab 6.0.1 (Bio-Rad #2012931).

### Hamster Infection Studies.

Male golden Syrian hamsters (3 to 4 wk old) were purchased from Envigo. All studies were conducted under a protocol approved by the UTMB Institutional Animal Care and Use Committee and complied with USDA guidelines in a laboratory accredited by the Association for Assessment and Accreditation of Laboratory Animal Care. Procedures involving infectious SARS-CoV-2 were performed in the Galveston National Laboratory ABSL3 facility. Animals were housed in groups of five and intranasally inoculated with 10^5^ pfu of WT or ΔQTQTN SARS-CoV-2. Animals were monitored daily for weight loss and development of clinical disease through the course of the study. Hamsters were anesthetized with isoflurane (Henry Schein Animal Health) for viral infection and nasal washes.

### Histology.

Left lungs were harvested from hamsters and fixed in 10% buffered formalin solution for at least 7 d. Fixed tissue was then embedded in paraffin, cut into 5 µM sections, and stained with hematoxylin and eosin on a SAKURA VIP6 processor by the University of Texas Medical Branch Surgical Pathology Laboratory.

### Structural Modeling.

Structural models were generated using SWISS-Model to generate homology models for WT, ΔQTQTN, and glycosylated QTQTN SARS-CoV-2 spike protein on the basis of the SARS-CoV-1 trimer structure (Protein Data Bank code 6ACD). Homology models were visualized and manipulated in PyMOL (version 2.4).

### Transcriptomics.

Hamster lungs were homogenized in TRIzol reagent (Thermo Fisher), and RNA was extracted with Direct-zol RNA Miniprep Plus kit (Zymo #R2072). The short-read sequencing libraries were generated from extracted RNA using Poly-A Click-Seq (PAC-Seq) (25, 26). Briefly, RNAs containing poly(A) tails were selectively reverse transcribed with oligo(dT) primers and stochastically terminated with azido-NTPS. Libraries were then gel purified (200-400 bp) and sequenced using Illumina platform (NextSeq550). Differential Poly-A Cluster (DPAC) was used to identify changes in overall expression (25, 26). A *P*-adjusted value (*P*-adj) of <0.1 and an absolute value of log2 fold change (|log2FC|) greater than 0.58 (or minimum of 50% increase/decrease) was used to filter results. The command ran for this data set was ∼/DPAC -p PMCDB -t 4 -x [flattened_annotations] -y [reference_names] -g [genome] -n 6 -v golden_hamster,Mesaur [metadata_file] [index] [experiment name] [output_directory], where -p indicates parameters used, in this case P (perform data preprocessing), M (map data), C (force new PAS cluster generation), D (perform differential APA analysis), and B (make individual bedgraphs), -t indicates how many threads were to be used, and -n indicates number of replicates. Annotations, gene names, index were used for Syrian golden hamster and mapped to the Syrian golden hamster genome (Mesaur). Data quality was determined to be sufficient by generating and loading bedgraph files into the UCSC Genome Browser (27).

### Nanoflow-LC-MS/MS Glycopeptide Mapping.

Sucrose cushion-purified SARS-CoV-2 virion samples were prepared for LC-MS/MS analysis as previously described (28). Samples were analyzed by nanoLC-MS/MS (nanoRSLC, ThermoFisher) using an Aurora series (Ion Opticks) reversed phase high-performance liquid chromatography column (25 cm length × 75 µm inner diameter) directly injected to an Orbitrap Eclipse using a 120 min gradient (mobile phase A = 0.1% formic acid (Thermo Fisher), mobile phase B = 99.9% acetonitrile with 0.1% formic acid (Thermo Fisher); hold 12% B for 5 min, 2 to 6% B in 0.1 min, 6 to 25% in 100 min, 25 to 50% in 15 min) at a flow rate of 350 nL/min. Eluted peptide ions were analyzed using a data-dependent acquisition (DDA) method with resolution settings of 120,000 and 15,000 (at *m/z* 200) for MS1 and MS2 scans, respectively. DDA-selected peptides were fragmented using stepped high energy collisional dissociation (27, 32, 37%). Tandem mass spectra were analyzed according to a label-free proteomic strategy using Proteome Discoverer (version 2.5.0.400, ThermoFisher) with the Byonic (version 4.1.10, Protein Metrics) and Minora nodes using specific single protein databases for each sample, respective to the individual Spike WT and mutant amino acid sequences (29, 30). Mass tolerances of 10 ppm and 20 ppm were used for matching parent and fragment masses, respectively. Mass spectra were searched with a fixed modification of carbamidomethyl (C), and up to two common variable modifications of deamidation (N,Q), oxidation (M), phosphorylation (S, T, Y), and glycan modifications using an *N*-glycan database of 182 human *N*-glycans, 1 maximum allowed; 70 human O-glycans, 1 maximum allowed. Peptide spectral matches were filtered for quality: PEP2D < 0.01, Byonic score > 100 (31).

## Supplementary Material

Supplementary File

## Data Availability

The raw [Transcriptomics] data have been deposited with links to NCBI BioProject databased under BioProject ID PRJNA856125 (32). The mass spectrometry proteomics data have been deposited to the ProteomeXchange Consortium via the PRIDE partner repository with the dataset identifier PXD034332 (33) and 10.6019/PXD034332 (34)
