# Supplementary material for: QTQTN motif upstream of the furin-cleavage site plays a key role in SARS-CoV-2 infection and pathogenesis
Source: Proc Natl Acad Sci U S A. 2022 Jul 26;119(32):e2205690119. doi: 10.1073/pnas.2205690119 (PMC9371735; doi:10.1073/pnas.2205690119)
Supplement: Supplementary File [file pnas.2205690119.sapp.pdf]

Supplemental Data
S. Figure 1-4
S. Table 1

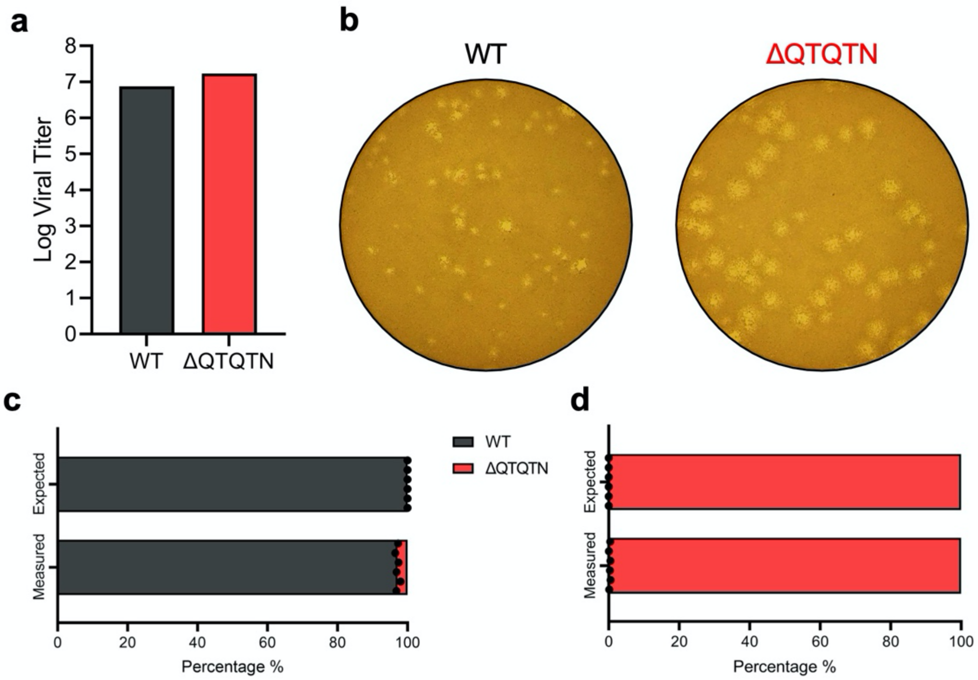

Supplemental Data Fig. 1: ΔQTQTN SARS-CoV-2 replication.
a, Virus stock titer of WT and ΔQTQTN SARS-CoV-2 from Vero E6. **b**, Plaque morphology of WT and ΔQTQTN in Vero E6. **c-d**, Competition assay between WT and ΔQTQTN SARS-CoV-2 at a ratio of 1:0 (**c**) and 0:1 (**d**) WT:ΔQTQTN, showing RNA percentage from next generation sequencing.

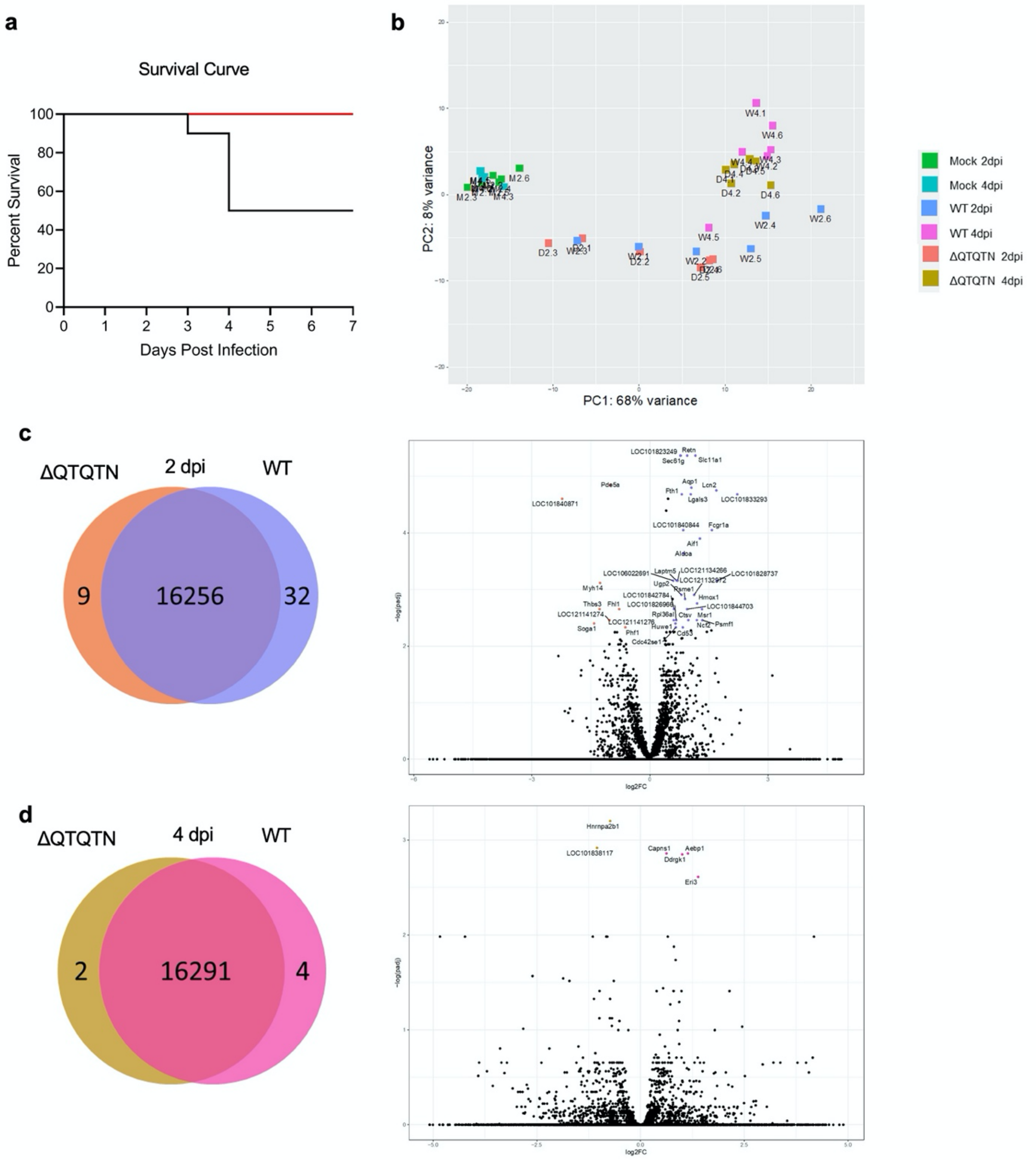

Supplemental Data Fig. 2: Hamster infection with ΔQTQTN SARS-CoV-2.
a, Survival curve (based on euthanasia criteria of >20% weight loss) following infection of WT or ΔQTQTN SARS-CoV-2 (n=10). **b**, Principal component analysis (PCA) plot of hamster lung samples. **c**, DESeq2 analysis of mapped genes between WT (purple) and ΔQTQTN (orange) at 2 dpi (left) with upregulated genes indicated in volcano plot (right). **d**, DESeq2 analysis of mapped genes between WT (purple) and ΔQTQTN (orange) at 4 dpi (left) with upregulated genes indicated in volcano plot (right).

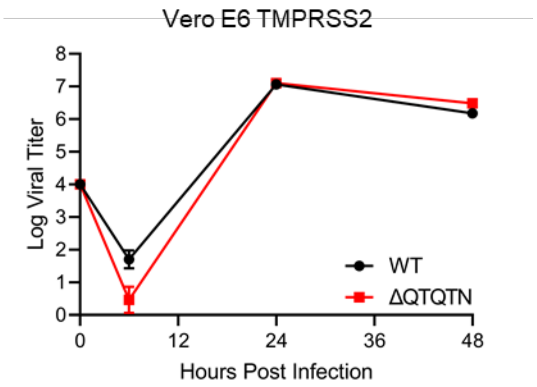

Supplemental Data Fig. 3: ΔQTQTN SARS-CoV-2 replication in TMPRSS2-expressing Vero E6. Viral titer from TMPRSS2-expressing Vero E6 infected with WT or ΔQTQTN SARS-CoV-2 at an MOI of 0.01 (n=3). Data are mean ± s.d. Statistical analysis measured by two-tailed Student's t-test. *, $p \leq 0.05$; **, $p \leq 0.01$; ***, $p \leq 0.001$; ****, $p \leq 0.0001$.

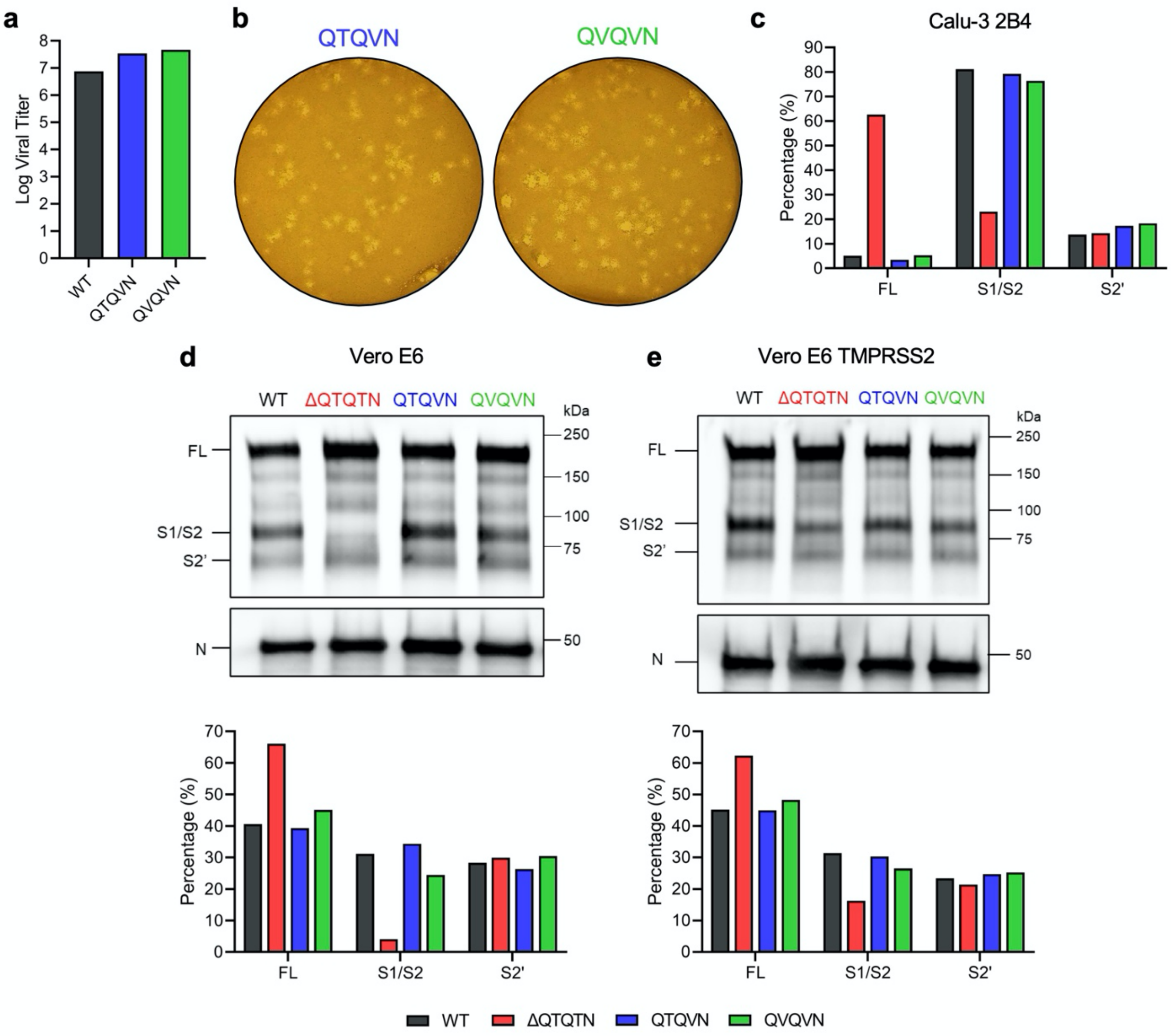

Supplemental Data Fig. 4: QTQVN and QVQVN SARS-CoV-2 replication and spike processing. **a**, Virus stock titer of WT, QTQVN, and QVQVN SARS-CoV-2 in Vero E6. **b**, Plaque morphology of QTQVN and QVQVN in Vero E6. **c**, Quantification by densitometry of full-length (FL), S1/S2 cleavage product, and S2’ cleavage product from western blot experiments of glycosylation mutants in Calu-3 2B4. **d-e**, Purified WT (black), ΔQTQTN (red), QTQVN (blue), and QVQVN (green) SARS-CoV-2 virions from Vero E6 (**d**) and TMRPSS2-expressing Vero E6 (**e**) probed with anti-S or anti-N antibody (upper). Full-length (FL), S1/S2 cleavage product, and S2’ cleavage product are indicated. Quantification of densitometry of FL, S1/S2, and S2’ normalized to N shown (lower). Results are representative of two experiments.

Virus	Coverage [%]	# Peptides	# Unique Peptides
SARS-CoV-2 WT	91	250	56
**Annotated Sequence**	**Modifications**	**Abundance**	
		**EThcD**	**HCD**
[R].AGCLIGAEHVNNSYECDIPIGAGICASYQTQTNSPR.[R]	Carbamidomethyl [C3; C16; C25]<br>Phospho [Y14]<br>HexNAc(2)Hex(4)Fuc(1) [N11]	4.31E+05	1.76E+06
[R].AGCLIGAEHVNNSYECDIPIGAGICASYQTQTNSPR.[R]	Carbamidomethyl [C3; C16; C25]<br>Deamidated [N12]<br>Phospho [Y14]<br>HexNAc(2)Hex(5)Fuc(1) [N11]	Not Detected	Below Quantitation Limit

Virus	Coverage [%]	# Peptides	# Unique Peptides
SARS-CoV-2 QTQVN	93	284	92
**Annotated Sequence**	**Modifications**	**Abundance**	
		**EThcD**	**HCD**
[R].AGCLIGAEHVNNSYECDIPIGAGICASYQTQVNSPR.[R]	3xCarbamidomethyl [C3; C16; C25]<br>1xPhospho [Y14]<br>1xHexNAc(2)Hex(3)Fuc(1) [N11]	Not Detected	5.61E+05
[R].AGCLIGAEHVNNSYECDIPIGAGICASYQTQVNSPR.[R]	3xCarbamidomethyl [C3; C16; C25]<br>1xDeamidated [N12]<br>1xPhospho [Y14]<br>1xHexNAc(2)Hex(3)Fuc(1) [N11]	Not Detected	5.61E+05
[R].AGCLIGAEHVNNSYECDIPIGAGICASYQTQVNSPR.[R]	3xCarbamidomethyl [C3; C16; C25]<br>1xPhospho [Y14]<br>1xHexNAc(2)Hex(5)Fuc(1) [N11]	3.51E+06	6.46E+06
[R].AGCLIGAEHVNNSYECDIPIGAGICASYQTQVNSPR.[R]	3xCarbamidomethyl [C3; C16; C25]<br>1xPhospho [Y14]<br>1xHexNAc(2)Hex(2)Fuc(1) [N11]<br>1xHexNAc(1)Hex(1)Fuc(1) [S13]	Not Detected	Below Quantitation Limit
[R].AGCLIGAEHVNNSYECDIPIGAGICASYQTQVNSPR.[R]	3xCarbamidomethyl [C3; C16; C25]<br>1xPhospho [Y]<br>1xHexNAc(2)Hex(1)Fuc(2) [S]<br>1xHexNAc(3)Hex(3) [S/T]<br>1xHexNAc(1)Hex(1)Fuc(1) [S13]<br>1xHexNAc(4)Hex(3)Fuc(1) [S27]	1.93E+06	1.24E+06
[R].AGCLIGAEHVNNSYECDIPIGAGICASYQTQVNSPR.[R]	3xCarbamidomethyl [C3; C16; C25]<br>1xPhospho [S13]<br>1xHexNAc(4)Hex(3)Fuc(1) [N11]<br>1xHexNAc(2)Hex(1)Fuc(1) [N11]<br>1xHexNAc(2)Hex(1)Fuc(2) [S27]<br>1xHexNAc(4)Hex(3)Fuc(2) [S27]	Below Quantitation Limit	Not Detected

Virus	Coverage [%]	# Peptides	# Unique Peptides
SARS-CoV-2 QVQVN	93	272	78
**Annotated Sequence**	**Modifications**	**Abundance**	
		**EThcD**	**HCD**
[R].AGCLIGAEHVNNSYECDIPIGAGICASYQVQVNSPR.[R]	3xCarbamidomethyl [C3; C16; C25]<br>1xPhospho [Y14]<br>1xHexNAc(2)Hex(4)Fuc(1) [N11]	5.49E+05	7.28E+05
[R].AGCLIGAEHVNNSYECDIPIGAGICASYQVQVNSPR.[R]	3xCarbamidomethyl [C3; C16; C25]<br>1xDeamidated [N12]<br>1xHexNAc(3)Hex(5) [N11]	1.21E+05	2.32E+05
[R].AGCLIGAEHVNNSYECDIPIGAGICASYQVQVNSPR.[R]	3xCarbamidomethyl [C3; C16; C25]<br>1xPhospho [Y]<br>1xHexNAc(2)Hex(5)Fuc(1) [N11]	3.28E+05	1.59E+06
[R].AGCLIGAEHVNNSYECDIPIGAGICASYQVQVNSPR.[R]	3xCarbamidomethyl [C3; C16; C25]<br>1xDeamidated [N12]<br>1xPhospho [Y14]<br>1xHexNAc(2)Hex(5)Fuc(1) [N11]	Not Detected	Below Quantitation Limit
[R].AGCLIGAEHVNNSYECDIPIGAGICASYQVQVNSPR.[R]	3xCarbamidomethyl [C3; C16; C25]<br>1xHexNAc(4)Hex(7)NeuAc(1) [N11]	Not Detected	Below Quantitation Limit

Supplemental Data Table S1. Quantification of Nanoflow-LC-MS/MS analysis of QTQTN glycosylation mutants.
Modifications and quantification of peptide spanning Spike 647-682 for WT, QTQVN and QVQVN
